# Identification of autism-related *MECP2* mutations by whole-exome sequencing and functional validation

**DOI:** 10.1186/s13229-017-0157-5

**Published:** 2017-08-03

**Authors:** Zhu Wen, Tian-Lin Cheng, Gai-zhi Li, Shi-Bang Sun, Shun-Ying Yu, Yi Zhang, Ya-Song Du, Zilong Qiu

**Affiliations:** 1 Shanghai Mental Health Center, Shanghai Jiao Tong University School of Medicine, Shanghai, China; 2 Institute of Neuroscience, CAS Center for Excellence in Brain Science and Intelligence Technology, Shanghai Institutes for Biological Sciences, Chinese Academy of Sciences, Shanghai, China; 3 School of Life Sciences, Peking University, Beijing, China; 4 Euler Genomics, Beijing, China

**Keywords:** Autism spectrum disorder, Methyl-CpG-binding protein-2 (MeCP2), Whole-exome sequencing, Neural development

## Abstract

**Background:**

Methyl-CpG-binding protein-2 (MeCP2) is a critical regulator for neural development. Either *loss-* or *gain-of-function* leads to severe neurodevelopmental disorders, such as Rett syndrome (RTT) and autism spectrum disorder (ASD). We set out to screen for *MECP2* mutations in patients of ASD and determine whether these autism-related mutations may compromise the proper function of MeCP2.

**Methods:**

Whole-exome sequencing was performed to screen *MECP2* and other ASD candidate genes for 120 patients diagnosed with ASD. The parents of patients who were identified with *MECP2* mutation were selected for further Sanger sequencing. Each patient accomplished the case report form including general information and clinical scales applied to assess their clinical features. Mouse cortical neurons and HEK-293 cells were cultured and transfected with MeCP2 wild-type (WT) or mutant to examine the function of autism-associated MeCP2 mutants. HEK-293 cells were used to examine the expression of MeCP2 mutant constructs with Western blot. Mouse cortical neurons were used to analyze neurites and axon outgrowth by immunofluorescence experiments.

**Results:**

We identified three missense mutations of *MECP2* from three autism patients by whole-exome sequencing: p.P152L (c.455C>T), p.P376S (c.1162C>T), and p.R294X (c.880C>T). Among these mutations, p.P152L and p.R294X were de novo mutations, whereas p.P376S was inherited maternally. The diagnosis of RTT was excluded in all three autism patients. Abnormalities of dendritic and axonal growth were found after autism-related MeCP2 mutants were expressed in mouse cortical neurons; suggesting that autism-related *MECP2* mutations impair the proper development of neurons.

**Conclusions:**

Our study identified genetic mutations of the *MECP2* gene in autism patients, which were previously considered to be associated primarily with RTT. This finding suggests that loss-of-function mutations of *MECP2* may also lead to autism spectrum disorders.

**Electronic supplementary material:**

The online version of this article (doi:10.1186/s13229-017-0157-5) contains supplementary material, which is available to authorized users.

## Background

Autism spectrum disorder (ASD) comprises a set of heterogeneous neurodevelopmental diseases characterized by early-onset difficulties in social communication/interaction and repetitive, restricted behavior/interests [1, 2]. According to a meta-analysis in China, the prevalence of autism is approximately 12.8 per 1000 individuals [3].

Methyl-CpG-binding protein 2 (MeCP2) is a critical regulator for brain development. Mutations of the *MECP2* gene lead to Rett syndrome (RTT), whereas duplications of *MECP2*-containing genomic segments cause the *MECP2* duplication syndrome [4, 5]. More than 95% of patients with RTT carry mutations in *MECP2* [6–9]. Mutations have also been reported in a few patients diagnosed with autism spectrum disorders [10–12]. However, whether *MECP2* mutations identified in patients with autism interfere with normal functions of MeCP2 remained unknown.

Regarding to clinical symptoms, there are some overlapping phenotypes between ASD and RTT, including social avoidance, stereotypic body/hand movement, and anxiety [13]. Furthermore, some girls with RTT also meet the criteria for ASD [14]. However, the core symptoms of RTT such as defects in locomotor activity and repetitive seizure are normally not often seen in ASD patients. There are severe autistic features identified in boys with the *MECP2* duplication syndrome [15].

In *Mecp2* null (*Mecp2* ^−/y^) mice, severe neurological phenotypic features, including hypoactivity, seizures, motor dysfunction, ataxia, and early death (8–10 weeks), were found [16, 17]. *MECP2* transgenic mice showed progressive neurological phenotypes including seizures, motor dysfunction, ataxia, and stereotypic behaviors [18]. Autistic-like behaviors (stereotypies and social behavior abnormalities) were also observed in *Mecp2* ^*308/Y*^ mouse models and transgenic monkeys overexpressing *MECP2* [19, 20].

In this study, we performed whole-exome sequencing on 120 ASD cases and identified three missense mutations in coding regions of the *MECP2* gene. The *MECP2* gene is associated with a host of neuropsychiatric disorders and neurological phenotypes. Moreover, *MECP2* mutations have been described in RTT, autism, mental retardation, and early-onset psychosis [11, 13, 21, 22]. We therefore hypothesized that mutations of *MECP2* in Chinese Han patients may be one of key contributors to ASD. We provided functional evidences and further suggested that genetic mutations associated with *MECP2* may shed light on the pathogenesis of autism and related neuropsychiatric disorders.

## Methods

### Ethics statement

We obtained assent from the Institutional Review Board (IRB), Shanghai Mental Health Center of Shanghai Jiao Tong University (FWA number 00003065, IROG number 0002202). Dr. Yi-Feng Xu approved and signed our study with ethical review number 2016–4. Written informed consent was obtained from parents in consideration of the fact that all patients were minors. All participants were screened using the appropriate protocol approved by the IRB.

### Subjects

A total of 120 core families with probands diagnosed with ASD were recruited from the outpatient Department of the Child and Adolescent Psychiatry, Shanghai Mental Health Center from 2013 to 2015. All patients were diagnosed on the basis of the fourth edition of the *Diagnostic and Statistical Manual of Mental Disorders* (DSM-IV) [23]. All patients were Han Chinese and their mean age was 78 months with ranged from 2 to 18 years. Among them, 102 were males and 18 were females. Any patient suffering a severe somatic disorder (including cardiomyopathy, tumor, and epilepsy) was excluded; those with a family history of psychosis were also excluded.

### Clinical scale assessment

For each patient, we collected a comprehensive profile including a detailed developmental history, clinical history, phenotypic features, family background, and scale examination. These were collected in the case report form (CRF). In CRF, all symptoms are classified into four parts in accordance with diagnostic items of ASD in DSM-IV: social interaction, language, repetitive behaviors, and functional impairment. Scales were applied to assess patients’ symptoms including Child Autism Rating Scale Score (CARS) and Autism behavior checklist (ABC). A cutoff score of CARS >36 represented severe autism, and a cutoff score of ABC >67 pointed to a high probability of autism.

### Whole-exome sequencing

#### DNA extraction and quality control of sample DNA

DNA was extracted from peripheral blood of patients and their parents using the DNeasy Blood &Tissue Kit (Cat no. 69506, QIAGEN, GmBH, Germany), following the manufacturer’s instructions. Qubit 2.0 Fluorometer (Cat no. Q32866, Invitrogen) was used to identify the quantity of purified DNA, and additional 1% agarose gel electrophoresis was performed to check the size integrity of purified DNA. Samples were excluded if the total mass, concentration, integrity of DNA, or quality of preliminary genotyping data was too low. Typical 2–3 μg genomic DNA was used for each WES experiments.

### Library preparation and assessment

For each sample to be sequenced, individual library preparations, hybridizations, and captures were performed following the protocol of the SureSelectXT Target Enrichment System for Illumina Paired-End Sequencing Library (Agilent Technologies, Inc., Santa Clara, CA) to assess quantity of library with Qubit 2.0 Fluoromete. The 2100 Bioanalyzer High Sensitivity DNA Assay was used to assess the quality and size range as instructed in the reagent kit guide.

### Preparation of libraries for cluster generation and sequencing

Libraries were quantified using qPCR (KAPA Biosystems) with probes specific to the ends of the adapters. The TruSeq PE Cluster Kit (Illumina) was used for cluster generation in an Illumina cBOT instrument following the manufacturer’s protocol (cBotTM User Guide). Libraries were loaded into each flow cell lane. Sequencing was performed on an Illumina HiSeq2500 instrument (Illumina) following the manufacturer’s protocol (HiSeq 2500 System User Guide). Multiplexed paired-read runs were carried out with 125 cycles.

### Reads mapping

The Reads (fastq files) were aligned to a human reference (hg19) by Burrows-Wheeler Aligner (BWA, v0.7.10). The aligned files (sam/bam format files) were sorted by samtools (0.1.19) first. Then, the aligned reads duplicating the start position of another read were flagged as duplicates (“Mark duplicate”) by using Picard Tools (1.107). Data were processed using the Genome Analysis ToolKit (GATK v3.1). Reads were locally realigned (GATK Indel Realigner) and their base qualities recalibrated (GATK Base Recalibrator). Finally, mapping statistics include coverage and depths were generated from recalibrated files by BEDTools (v2.16.1) and perl/python scripts.

### Variant calling and annotation

Variants (SNVs and indels) were identified and genotyped from recalibrated BAM files using the multisample processing mode of the Unified Genotyper tool from the GATK. Then, we use the variant quality score recalibration (VQSR) training sets method (ref: https://www.broadinstitute.org/gatk/guide/topic?name=methods) and the resource datasets and arguments that GATK recommends to generate the significantly confident variants. Variants (SNVs and indels) were annotated on the basis of the hg19 database using ANNOVAR software (ref:http://annovar.openbioinformatics.org/en/latest/user-guide/download/) and the GATK Variant Annotator. The databases used to annotate were as follows: reGene, sift, 1000G, dbSNP138, COSMIC, OMIM, ClinVar, SIFT, and Polyphen-2.

### Variant filters and quality control

First, we performed several quality control (QC) steps to identify and remove low-quality variants. We required that QC of Variations Calling were judged as “PASS”-based on Variant Recalibrator and Apply Recalibration of GATK, coverage was over 90%, and reads on target region (%) was over 94%. Second, we required that variant location at exon or splicing site. Third, we selected exonic Function including non-synonymous SNV, stopgain/loss, startgain/loss splice site, and frameshift. Fourth, MAF ≤ 1% or null in 1000G-ASN. Finally, the results of primary analysis by polyphen-2, SIFT, GERP++, ClinVar, and MA also are taken into account.

### Variation identify by Sanger sequencing

Based on the data from WES, all families within probands carrying MECP2 variations were selected for Sanger sequencing to validate whether variations are de novo or inherited from parents. The primers for Sanger sequencing are listed in Additional file 1: Table S1.

### Experiment of molecular biology

#### Plasmids

The MeCP2-WT gene (the E2 isoform of rat *Mecp2* cDNA) was a gift from Dr. Adrian Bird. Other plasmids of MeCP2 mutations and truncations detected from ASD probands were generated on the construct. MeCP2-P152L, MeCP2-R294X, and MeCP2-P376S were generated using KOD-mutagenesis (Toyobo). The primers desired for generating mutant were list below (Table 1).

**Table 1:** Summary of variations in ASD patients carrying *MECP2* mutations

Patient ID	Sex	Gene	Chrom	Position (Hg19)	Nucleotide	Amino acid	1000G	dbSNP137	Transmission
138	F	MECP2	ChrX	153,296,824	c.455C>T	p.P152L	_	_	De novo
		NRXN1	Chr2	50,280,477	c.4180A>T	p.T1394S	0.00079	rs202006815	Father
		CACNA1C	Chr12	2,789,681	c.5564G>A	p.C1855Y	_	_	Father
548	M	MECP2	ChrX	153,296,153	c.1162C>T	p.P376S	0.00026	rs61752387	Mother
		CNTNAP2	Chr7	146,536,803	c.209G>C	p.G70A	_	_	Father
660	F	MECP2	ChrX	153,296,399	c.880C>T	p.R294X	_	rs61751362	De novo

*Abbreviation*: *F* stands for female, *M* stands for male

#### Antibodies

Antibodies used in this study were as follows: rabbit-anti-GFP (#G10362, Invitrogen), SMI312 (Convance), 488-rabbit (Invitrogen), 555-mouse (Invitrogen), DAPI (DAPI), mouse-anti-HA epitope tag (#M20013; Abmart), HRP-goat-anti-mouse (GE), and GAPDH (ab8245; Abcam).

#### Cell culture

Embryonic days 15–16 (E15-E16) of mouse (C57B/L6) cortical neurons and HEK-293 cells were cultured and transfected each group via electrophoretic transfer at 0 days in vitro (DIV) separately. One or two plasmids (fugw-GFP, fugw-wild-type-GFP, fugw-GFP: fugw-mutant = 1:3) were co-transfected Amaxa Nucleofector in 6-well plates or 12-well plates covered with glasses coated with a poly-D-lysine (PDL) solution (1 mg/mL). After 3 DIV, HEK-293 cells in 6-well plates were collected for Western blot, and mouse cortical neurons on the glasses of 12-well plates were fixed for further immunofluorescence analysis.

#### Western blot

Briefly, the liquid culture medium was removed first, proteins were isolated via 1× loading buffer (SDS) from 6-well plates (300 ul/well) and then heating at approximately 100 °C for 20 min via metal bath. Proteins were run on upper gel (60 v for 60 min) and separation gel (120 v for 90 min) by turn and then transferred to PVDF membrane in SDS-PAGE gel (200 mA for 70 min). The PVDF membrane was blocked in 5% BSA for 3 h. Immunoblotting was performed using the primary antibodies (1:10,000) at 4 °C overnight. After being washed three times by 1× TBST, secondary antibodies were conjugated to MeCP2-WT and MeCP2-mutant and detected by chemiluminescence. MeCP2 and GAPDH protein levels were quantified using ImageJ software.

#### Immunofluorescence

For calculating neurites and axon outgrowth of neurons, they were washed with 1× PBS for 5 min, fixed in 4% PFA for 20 min and blocked in 1× PBS buffer with 3% BSA and 0.1% Triton X-100 for 60 min at room temperature (RT). The cells were incubated in primary antibodies overnight at 4 °C, washed three times in 1× PBS for 60 min and then incubated in secondary antibodies at RT for 90 min. The target neurons were marked with GFP signals observed via fluorescence microscopy.

#### Analysis of dendrites and axons

In at least three independent experiments performed, about 40–50 GFP-positive neurons were picked up randomly from each group. The searcher was blinded until statistical analysis was completed. Then, the images were analyzed using Fiji software according to the standard: all of dendritic branches and secondary branch, the longest axon (exclude sub-axon), and the total length of all neurites were taken into account respectively. All measurement data were examined with a Student’s *t* test, and significant level was considered if *p* < 0.05.

#### Web sources

dbSNP database (http://www.ncbi.nlm.nih.gov/projects/SNP/index.html).

1000 Genomes Project data (http://www.1000genomes.org/)

RettBASE (http://www.rettsyndrome.org/)

GnomAD (http://gnomad.broadinstitute.org/)

## Results

### Whole-exome sequencing

**Fig. 1:**
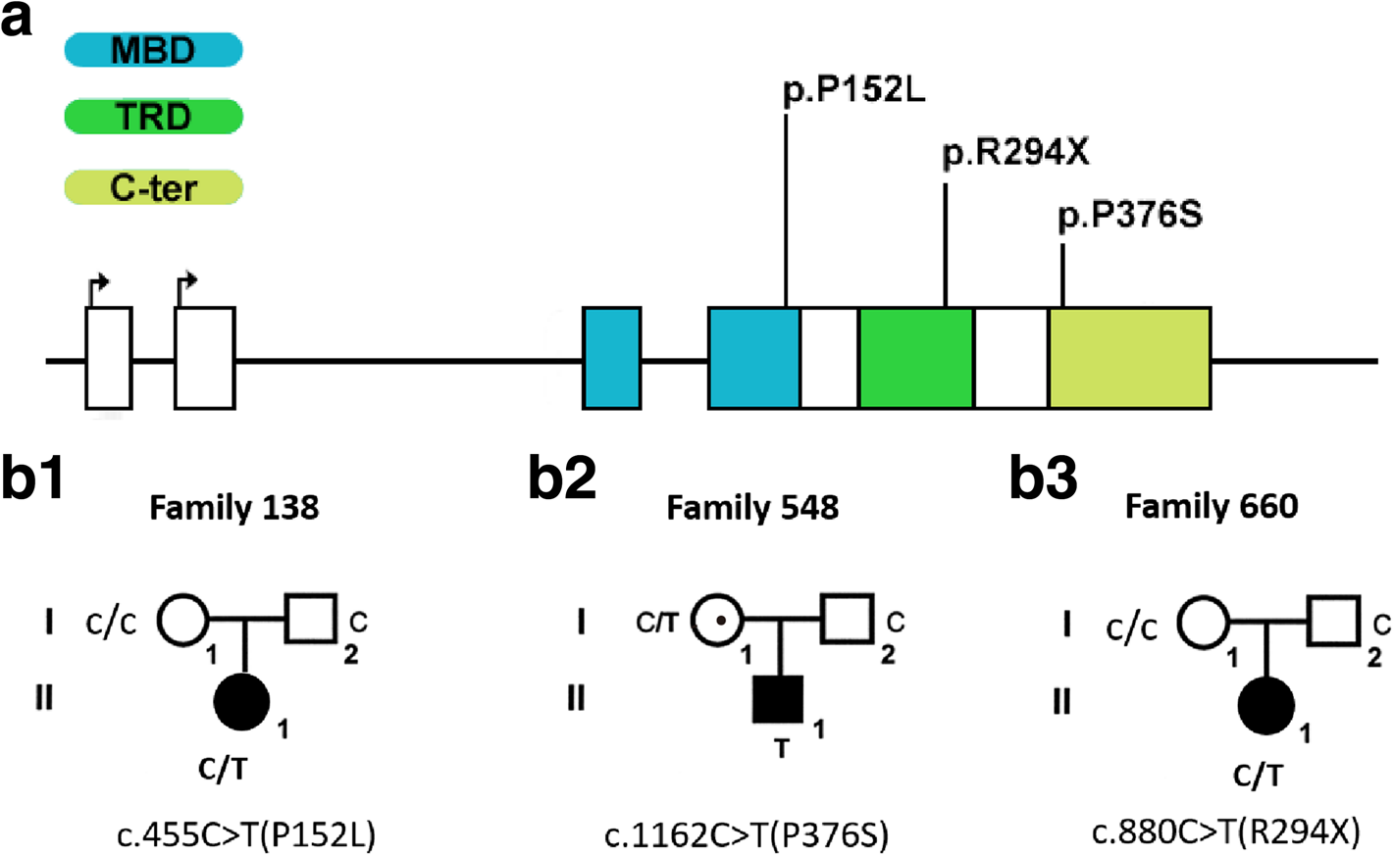
The location of mutations on the *MECP2* gene and genetic genogram of the three core families. **a** The location of three *MECP2* mutation detected from ASD patients. p.P152L locates on exon 2, MBD; p.P376S locates on exon 3, TRD, p.R294X locates on exon 4, C-ter. **b** Genogram of the three families within proband carrying *MECP2* mutation

Among 120 ASD patients, three mutations of *MECP2* were detected from three probands via WES: p.P152L (c.455C>T), p.P376S (c.1162C>T) were missense mutations, and p.R294X (c.880C>T) was truncating mutation (NCBI accession number NM_004992). Furthermore, Sanger sequencing was performed to confirm these mutants and identify they were inherited or de novo. We found that p.P152L and p.R294X were de novo mutations; however, p.P376S was inherited maternally (Fig. 1, Table 1). The basic information of *MECP2* mutations was summarized in Table 1 and the genogram of the families showed in Fig. 1.

Further analysis by searching dbSNP database, 1000 Genomes Project data, and RettBASE, p.P152L have not been reported previously; p.R294X (rs61751362) and p.P376S (rs61752387) have frequently been reported in RTT patients. The mutation frequency of p.P376S is 0.00026 in 1000G (Table 1). We also search these mutations in GnomAD database and found that none of these mutants existed in GnomAD database, indicating that they are extremely rare mutations.

**Fig. 2:**
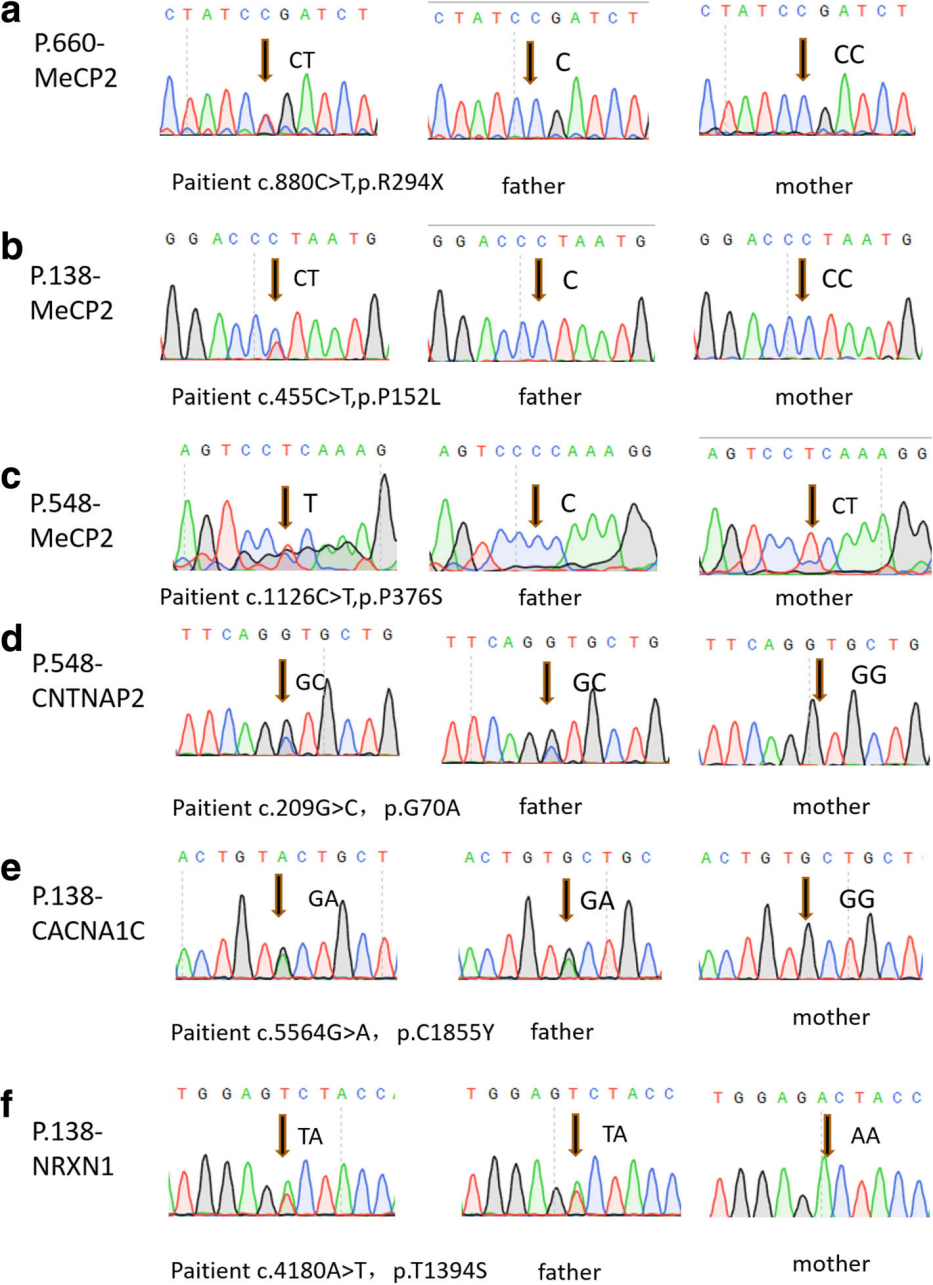
Sanger sequencing diagram of MECP2 and other genes variations in autism core family. **a** P.660 showed heterozygous mutation c.455C>T of *MECP2*, parents indicated homozygote (CC/C). **b** P.138 showed heterozygous mutation c.880C>T of *MECP2*, parents indicated homozygote (CC/C). **c** P.548 showed heterozygous mutation c.1126C>T of *MECP2*, proband’s father indicated homozygote (C), but proband’s mother indicated heterozygote (CT). **d** P.548 showed heterozygous mutation c.209G>C of *cntnap2*, proband’s mother indicated homozygote (GG), but proband’s father indicated heterozygote (GC). This mutation of P.548 was inherited from his father. **e** P.138 showed heterozygous mutation c.5564G>A of *CACNA1C*, proband’s mother indicated homozygote (GG), but proband’s father indicated heterozygote (GA). This mutation of P.138 was inherited from his father. **f** P.138 showed heterozygous mutation c.4180A>T of NRXN1, proband’s mother indicated homozygote (AA), but proband’s father indicated heterozygote (AT). This mutation of P.138 was inherited from his father

We also detected variants of other ASD candidate genes on the three carriers with *MECP2* mutation. We found p.T1394S (c.4180A>T) of *NRXN1* and p.C1855Y (c.5564G>A) of *CACNA1C* on patient 138 (P.138) and p.G70A (c.209G>C) of *CNTNAP2* on P.548. Although these genes are also candidate genes for ASD, all the heterozygotes were inherited from unaffected fathers according to data from Sanger sequencing (Table 1, Fig. 2).

### Clinical features of *MECP2* mutation carriers

Overall, poor social interaction was highlighted symptom, but language using and repetitive behavior differ greatly in these three patients. Functional impairment was outstanding in daily life and learning for all the patients. It is worth noting that all of them were excluded in the diagnosis of RTT (RTT symptoms are negative, such as loss of hand skills, seizures, motor abnormalities).

In CRF, P.138 matched all items of social and language (full items = 4), however, just satisfied one item of stereotyped behavior (full items = 4). P.548 showed symptoms in all parts, social (matched items = 3), language (matched items = 3), and repetitive behaviors (matched items = 4). For P.660, the main symptom was social disorder (matched items = 4), but language (matched items = 1) and stereotyped behavior (matched items = 1) were relatively mild (Table 2).

**Table 2:** Summary of the phenotypic features of ASD patients carrying *MECP2* mutations

Subject	P.138	P.548	P.660
Score on CARS	30	48	53
Score on ABC	158	245	195
Matched items in DSM-IV	12	13	9
Social (4)	4	3	4
Language (4)	4	3	1
Stereotypic behavior (4)	1	4	1
Functional impairment (3)	3	3	3
Dyslexia	Y	Y	Y
Mathematics disorder	Y	Y	Y
Brain imaging scan	NT	N	Y
Comorbidity	ADHD	N	Mental retardation
Family history	N	N	N

*Abbreviations*: *Y* yes, *NT* not test, *N* normal, *NA* information not available, *CARS* Child Autism Rating Scale Score, *ABC* autism behavior checklist

The score of child autism rating scale (CARS) of P.138, P.548, P.660, was 30, 48, 53, respectively, (full mark = 60, cutoff of diagnosing autism ≥30, cutoff of severe autism ≥36). Therefore, the scores indicating all of them achieved standard of diagnosing autism.

From the results of Autism Behavior Checklist (ABC), the score of P.138, P.548, P.660, was 158, 245, and 195, respectively, (the cutoff of screening autism >56, the cutoff of diagnosing autism >61). Therefore, the results manifested all of them were also above diagnostic score of autism, and the higher score indicated more severe autistic symptoms such as P.548 and P.660.

In addition, 70% of ASD patients with various comorbidity, and in our patients, P.138 appeared attention deficit and hyperactivity according to descriptions of parents, but she was not diagnosed with attention deficit hyperactivity disorder (ADHD). P.548 does not have comorbidities at present. P.660 has mental retardation (MR). None of the three patients have familial history of psychiatric disorders or neuropathy.

### Developmental history and family background of carriers of the *MECP2* mutation

ASD symptoms of P.138 (female) were onset at age 3 and diagnosed at age 5. Her mother suffered preeclampsia at 3–7 months during pregnancy. Her developmental history indicated she spoke first word at 7 months, started walking by herself at 13 months. Her personality characteristics manifested sensitivity, anxiety, and irritability, indifference according to parents’ description (Additional file 2: Table S2).

P.548 (male), onset before 2 years of age, was diagnosed at 4 years of age. His mother had influenza during her pregnancy, and he did not receive breastfeeding. His developmental history indicated he spoke first word at 26 months (indicating language developmental delay), started walking independently at 16 months (indicating possible motor development delay). Her personality characteristics manifested sensitive based on parents’ description (Additional file 2: Table S2).

P.660 (female) onset at 32 months was diagnosed at 38 months. Her mother was diagnosed by amniotic fluid pollution before delivery with her gestational weeks >40 weeks. Her developmental history indicated she spoke first word at 10 months, started walking by herself at 14 months. Her personality characteristics manifested indifference according to parents’ description. More details about these patients are showed in Additional file 2: Table S2.

### Functional analysis of autism-related *MECP2* mutations

**Fig. 3:**
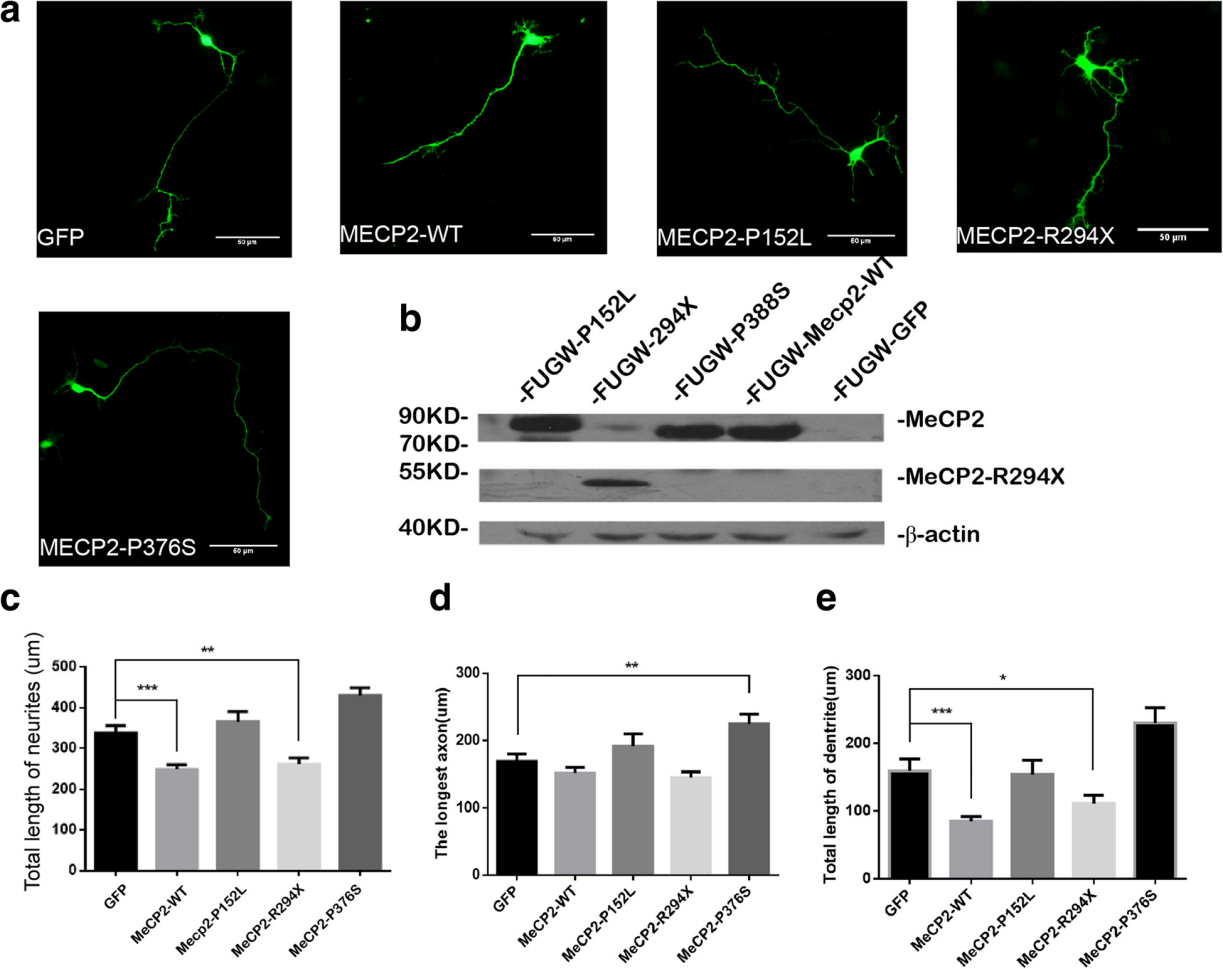
Results of Western blot and immunofluorescence from MeCP2 WT and mutant expression in vitro. **a** The example picture of mouse primary cortical neurons transfected (0 DIV) with GFP alone and GFP with constructs MECP2 mutant or wild type at 3 DIV. “Total length of neurons,” which means the length of all dendritic branches (including secondary branch) add the axon (include sub-axon). **b** Identification of MeCP2 expression by Western blots in each condition: GFP (null), MeCP2-WT (70~100 KD), MeCP2-P152L (70~100 KD), MeCP2-R294X (40~55 KD), MeCP2-P376S (70~100 KD), β-actin (≈40 KD). **c**–**e** The length of total neurites, the longest axon, and total dendrite of each condition. *p < 0.05, **p < 0.01, ***p < 0.001 (*t* test). All neurons were fixed at 3 DIV and immunostained with GFP antibody for measurement. A total 40–50 neurons from each condition were randomly selected and measured via blind method

To determine whether these autism-related *MECP2* mutations may affect the proper function of the MeCP2 protein, we performed experiments in culture mouse primary neurons by expressing GFP plasmids together with each *MECP2* mutants and wild-type MeCP2 (Fig. 3a, Additional file 3: Figure S1). We also tested the expression of GFP, MeCP2-WT, MeCP2-P152L, MeCP2-R294X, and MeCP2-P376S in HEK-293 cells by Western blots (Fig. 3b).

Next, we measured the neurite length in neurons transfected with various constructs. First, we found that overexpression of MeCP2-WT indeed inhibited dendritic growth as previously reported [25]. However, MeCP2-P152L and MeCP2-P376S mutants have no effects on dendritic growth, suggesting that they are loss-of-function mutations (Fig. 3c, e). Moreover, overexpression of MeCP2-P376S appears to increase the axonal length, suggesting that MeCP2-P376S may be gain-of-function, in term of axonal development (Fig. 3d). Abnormal axonal development likely causes defects neural development in patients.

## Conclusions

Taken together, these lines of evidence strongly suggest that MeCP2-P152L, MeCP2-R294X, and MeCP2-P376S, identified in autism patients, affect the proper physiological functions of the MeCP2 protein and thus may contribute the pathogenesis of autism.

## Discussion

In this work, we found that three rare variants of *MECP2* were detected in three patients by WES: c.455C>T, p.P152L; c.1162C>T, p.P376S; and c.880C>T, p.R294X. Furthermore, we detected two of the three also carried variants of other genetic mutations (P.138: NRXIN1 c.4180A>T, CACNA1C c.5564G>A; P.548: CNTNAP2, c.209G>C). We identified two *MECP2* variations (p.P152L and p.R294X) were de novo mutations by Sanger sequencing, and all probands have no familial history of psychiatry and neuropathy. Therefore, these mutations in *MECP2* were highly possible to contribute to clinical phenotypes.

Clinically, all 120 patients participating in this study were diagnosed with ASD/autism strictly according to DSM-IV (Additional file 4). The three patients carrying *MECP2* mutations were typical autism. First, the age of onset were before 3 years (36 months), P.548 had developmental delay in language. Second, the scores of ABC and CARS were over the diagnostic score of autism which were corresponding to the diagnosis by psychiatrists. Third, core symptoms existed, including difficulties in reciprocal social interaction and communication (limited shared enjoyment in interactions, limited range of facial expression, and eye gaze), repetitive patterns of behavior/interests (playing the wheel of toy car for 1–2 h persistently). In addition, among these *MECP2* mutations, p.P152L is located on the crucial methyl-DNA-binding domain (MBD) and have not been reported, although the mutations (P152R, P152A) on the same site of *MECP2* have described in patients with RTT. It was noteworthy that p.R294X and p.P376S were also detected in patients with atypical or classical RTT, ASD, and mental retardation previously [10, 11]. First, p.R294X is common and severe mutation in patients with RTT [24]. Moreover, p.R294X is located on the transcriptional repression domain (TRD), which lead to incomplete TRD and deleted the whole C-terminal. The girl with p.R294X (P.660) also showed mild mental retardation. p.R294X was also reported as disease-causing mutation in Asian population, in which two patients carrying p.R294X diagnosed with RTT exhibited features including developmental delay and difficulties in walking and speaking, and abnormal respiration [25]. Consistent with the patient P.660, a female with a de novo mutation p.R294X matches the DSM-IV criteria for autism and confirmed by the ADI-R [11].

p.P376S was also found in female patients with classical RTT [9], one of studies reported a boy and his mother carrying p.P376S were both diagnosed with autism [10]. Since *MECP2* is the dosage-sensitive gene and closely related to epigenetic regulation with lots of genes involved in neurodevelopment and neuronal functional integrity [15], it is possible that the milder phenotype in our cases with autism may be due to genetic modifiers. We prefer the possibility that the mild skewing of X chromosome inactivation (XCI) partially alleviates the consequences of the *MECP2* mutations. Therefore, the findings in the three probands with autism indicated that the phenotypic heterogeneity of *MECP2* mutations is quite variable.

Previous work suggested that the development of proper dendritic morphology needs a precise set point of MeCP2 expression, either the overexpression or the elimination of MeCP2 results in a decrease in dendritic arbor [26]. Another study also found that overexpression of MeCP2-WT inhibited dendritic growth in hippocampal slice cultures [27]. Results in the previous study showed overexpression of MeCP2-mutant (MeCP2^380^ and MeCP2^380A^) had no effect on dendritic growth, in consistent with the fact that overexpression of MeCP2-P152L and MeCP2-P376S made no difference on the dendritic growth comparing with control. In addition, overexpression of MeCP2-P376S likely promotes the outgrowth of axon, suggesting that P376S is gain-of-function mutation. The results suggested the mutations of *MECP2* found in ASD patients were closely associated with neurodevelopment.

Taken together, patients with the various mutations of *MECP2* show a broad array of phenotypes. This study highlights the vital role of MeCP2 in the neural development. Therefore, it is necessary to scan the *MECP2* as one of candidate genes in ASD patients. Further work is needed to clarify functions of these mutations to neural development in animal models.

## Additional files

Additional file 1: Table S1. Primers applied for Sanger sequencing and mutagenesis. (DOCX 14 kb)

Additional file 2: Table S2. Summary of developmental and family status of ASD patients carrying *MECP2* mutations. (DOCX 16 kb)

Additional file 3: Experiments in culture mouse primary neurons by expressing GFP plasmids together with each *MECP2* mutants and wild-type MeCP2. (PNG 1144 kb)

Additional file 4: Table S3. Features of all 120 participants with ASD. (DOCX 29 kb)

## Abbreviations

ABC: Autism Behavior Checklist
ADI-R: Autism diagnostic interview-revised
ASD: Autism spectrum disorder
CARS: Child Autism Rating Scale score
CDD: Childhood disintegrative disorder
DSM-IV: Diagnostic and statistical manual of mental disorders fourth edition
MPS IIIA: Mucopolysaccharidosis IIIA
MRI: Magnetic resonance imaging
PDD: Pervasive developmental disorder
uGAG: Urinary glycosaminoglycan
WES: Whole-exome sequencing
