# Supplementary material for: Identification of autism-related MECP2 mutations by whole-exome sequencing and functional validation
Source: Mol Autism. 2017 Aug 3;8:43. doi: 10.1186/s13229-017-0157-5 (PMC5543534; doi:10.1186/s13229-017-0157-5)
Supplement: Supplementary file 1 — Primers applied for Sanger sequencing and mutagenesis. (DOCX 14 kb) [file 13229_2017_157_MOESM1_ESM.docx]

**Table S1. Primers Applied for Sanger Sequencing and Mutagenesis**

| Primers | Sequencing (5' – 3') |
| --- | --- |
| MECP2-138-f | cgtttgtcagagcgttgtca |
| MECP2-138-r | AGGCATCTTGACAAGGAGCT |
| MECP2-548-f  MECP2-548-r | GGTGGAGGTGGGGGCAGG  CTCGGTGAGAAGAGCGGG |
| MECP2-660-f | CCACATCCACCCAGGTCA |
| MECP2-660-r | CAGGGCTCTTACAGGTCTTCA |
| CACNA1C-138-f | tcctctcatccctgtgctct |
| CACNA1C-138-r | catgcagccctcttgaaatg |
| NRXN1-138-f | GCGTCAGCTCACAATCTTCA |
| NRXN1-138-r | tgcagaaaagcccactatca |
| CNTNAP2-548-f | gttgaagtcccaatggcatc |
| CNTNAP2-548-r | gtctttgctttcctgccaat |
| CBS-548-f | cccgagtggacacagaaact |
| CBS-548-f | gtggcaggatggagaggag |
| MECP2-mut-138-f | CTTAATGATTTTGACTTCACGGTAA |
| MECP2-mut-138-r | GTCCAGGGATGTGTCGCCTA |
| MECP2-mut-660-f | TGATCTGTGCAGGAGACCGTA |
| MECP2-mut-660-r | GATAGAAGACTCCTTCACGGCT |
| MECP2-mut-548-f | TCAAAGGCCCCCGTGCCACT |
| MECP2-mut-548-r | GGACTCTGAGTGGTGGTGATGGTGG |
