# Supplementary material for: Identification of autism-related MECP2 mutations by whole-exome sequencing and functional validation
Source: Mol Autism. 2017 Aug 3;8:43. doi: 10.1186/s13229-017-0157-5 (PMC5543534; doi:10.1186/s13229-017-0157-5)
Supplement: Supplementary file 2 — Summary of developmental and family status of ASD patients carrying MECP2 mutations. (DOCX 16 kb) [file 13229_2017_157_MOESM2_ESM.docx]

**Table S2. Summary of Developmental and Family Status of ASD Patients Carrying *MECP2* Mutations**

| **Subject** | P.138 | P.548 | P.660 |
| --- | --- | --- | --- |
| **gender** | female | male | female |
| **Age at first episode** | 36 months | 21 months | 32 months |
| **Age at diagnosis** | 60 months | 48 months | 38 months |
| **History of pregnancy and delivery** |  |  |  |
| Age of parents at time of pregnancy | <30 | <30 | <30 |
| Diseases during pregnancy | preeclampsia (at 3–7 months) | influenza | amniotic fluid pollution |
| Order of pregnancy | first | second | NA |
| Birth order | first | first | NA |
| Gestational weeks | 38–40 | 38–40 | >40 |
| Breastfeeding | Y | N | Y |
| **Developmental history** |  |  |  |
| Age at first word | 7 months | 26 months | 10 months |
| Start of walking | 13 months | 16 months | 14 months |
| Personality characteristics | sensitive, anxious,  irritable, apathic | sensitive | apathic |
| **Family background** |  |  |  |
| Income per person per month | 500–1000 yuan | 1000–3000 yuan | >10,000 yuan |
| Relationship of parents | NA | inharmonious | harmonious |
| Family type | core family | core family | three-generation family |
| **Parenting status** |  |  |  |
| Parenting pattern between parents | inconsistent | inconsistent | consistent |
| Parenting style | beating and scolding, rigorous constraints | beating and scolding | permissive and spoiling |
| Time with parents | <1 hour | <1 hour | >3 hours |
| Parents' impression of feeding | difficult | difficult | difficult |

Abbreviations: Y: Yes; N:normal；NA：information not available;
