# Supplementary figures and images for: Identification of autism-related MECP2 mutations by whole-exome sequencing and functional validation
Source: Mol Autism. 2017 Aug 3;8:43. doi: 10.1186/s13229-017-0157-5 (PMC5543534; doi:10.1186/s13229-017-0157-5)

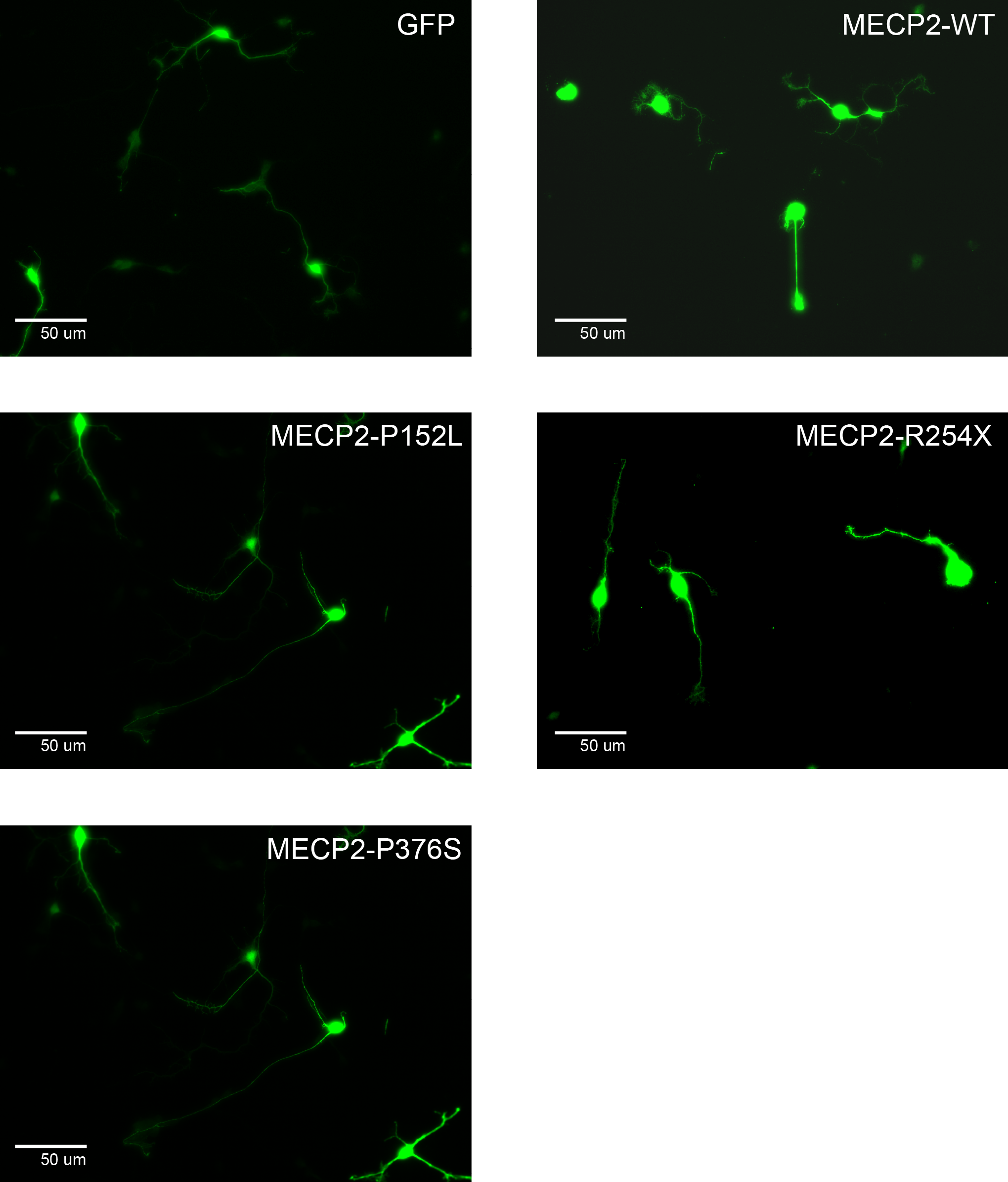

Supplement: Supplementary file 3 — Experiments in culture mouse primary neurons by expressing GFP plasmids together with each MECP2 mutants and wild-type MeCP2. (PNG 1144 kb) [file 13229_2017_157_MOESM3_ESM.png]
