# Supplementary material for: Identification of autism-related MECP2 mutations by whole-exome sequencing and functional validation
Source: Mol Autism. 2017 Aug 3;8:43. doi: 10.1186/s13229-017-0157-5 (PMC5543534; doi:10.1186/s13229-017-0157-5)
Supplement: Supplementary file 4 — Features of all 120 participants with ASD. (DOCX 29 kb) [file 13229_2017_157_MOESM4_ESM.docx]

Table5. Features of all 120 participants with ASD

| PatientID | Gender（1=male，2=female） | Score of ABC | Social (4) | Language (4) | Stereotypic behavior(4) | Functional impairment (3) | DSM-IV |
| --- | --- | --- | --- | --- | --- | --- | --- |
| 56 | 2 | 144 | 4 | 2 | 3 | 3 | 12 |
| 138 | 2 | 158 | 4 | 4 | 1 | 3 | 12 |
| 141 | 2 | 140 | 4 | 3 | 3 | 1 | 11 |
| 142 | 1 | 117 | 4 | 4 | 0 | 2 | 10 |
| 143 | 1 | 150 | 3 | 3 | 3 | 3 | 12 |
| 145 | 1 | 86 | 3 | 3 | 1 | 3 | 10 |
| 148 | 2 | 85 | 3 | 4 | 3 | 3 | 13 |
| 152 | 1 | 160 | 4 | 2 | 0 | 3 | 9 |
| 163 | 1 | 176 | 4 | 4 | 4 | 3 | 15 |
| 165 | 1 | 135 | 3 | 2 | 3 | 3 | 11 |
| 172 | 1 | 144 | 3 | 4 | 2 | 3 | 12 |
| 173 | 1 | 97 | 2 | 2 | 0 | 2 | 6 |
| 175 | 1 | 119 | 2 | 3 | 0 | 1 | 6 |
| 176 | 1 | 103 | 3 | 2 | 0 | 3 | 8 |
| 180 | 1 | 106 | 4 | 3 | 1 | 3 | 11 |
| 181 | 1 | 82 | 2 | 1 | 0 | 3 | 6 |
| 182 | 1 | 117 | 3 | 3 | 2 | 3 | 11 |
| 183 | 1 | 94 | 2 | 2 | 2 | 0 | 6 |
| 190 | 1 | 154 | 3 | 2 | 3 | 3 | 11 |
| 191 | 1 | 152 | 3 | 3 | 4 | 2 | 12 |
| 192 | 1 | 143 | 3 | 4 | 3 | 3 | 13 |
| 194 | 1 | 140 | 1 | 3 | 2 | 2 | 8 |
| 197 | 1 | 128 | 2 | 2 | 3 | 1 | 8 |
| 199 | 1 | 89 | 3 | 2 | 2 | 2 | 9 |
| 200 | 1 | 176 | 3 | 3 | 2 | 3 | 11 |
| 207 | 2 | 149 | 4 | 4 | 1 | 3 | 12 |
| 329 | 1 | 99 | 2 | 3 | 3 | 3 | 11 |
| 335 | 1 | 116 | 2 | 3 | 1 | 3 | 9 |
| 337 | 2 | 71 | 1 | 1 | 1 | 3 | 6 |
| 341 | 1 | 97 | 4 | 2 | 1 | 2 | 9 |
| 354 | 1 | 75 | 3 | 2 | 2 | 1 | 8 |
| 366 | 1 | 137 | 3 | 3 | 2 | 3 | 11 |
| 375 | 1 | 210 | 3 | 4 | 3 | 3 | 13 |
| 377 | 1 | 146 | 3 | 2 | 2 | 3 | 10 |
| 393 | 1 | 160 | 4 | 3 | 1 | 3 | 11 |
| 394 | 2 | 84 | 2 | 3 | 0 | 3 | 8 |
| 397 | 1 | 169 | 3 | 4 | 1 | 3 | 11 |
| 469 | 1 | 96 | 3 | 3 | 3 | 2 | 11 |
| 490 | 1 | 102 | 2 | 3 | 2 | 2 | 9 |
| 507 | 1 | 79 | 2 | 3 | 1 | 2 | 8 |
| 523 | 1 | 240 | 4 | 3 | 4 | 3 | 14 |
| 548 | 1 | 245 | 3 | 3 | 4 | 3 | 13 |
| 645 | 1 | 178 | 3 | 4 | 4 | 3 | 14 |
| 647 | 1 | 76 |  |  |  |  |  |
| 648 | 2 | 137 | 3 | 2 | 2 | 3 | 10 |
| 652 | 1 | 130 | 4 | 2 | 1 | 1 | 8 |
| 653 | 2 | 160 |  |  |  |  |  |
| 655 | 1 | 131 |  |  |  |  |  |
| 660 | 2 | 195 | 1 | 2 | 3 | 4 | 10 |
| 661 | 1 | 243 | 3 | 4 | 4 | 3 | 14 |
| 664 | 1 | 157 | 4 | 4 | 2 | 2 | 12 |
| 673 | 1 | 149 | 4 | 2 | 0 | 3 | 9 |
| 682 | 1 | 128 |  |  |  |  |  |
| 686 | 1 | 102 | 2 | 2 | 1 | 1 | 6 |
| 687 | 2 | 129 | 3 | 2 | 2 | 3 | 10 |
| 700 | 1 | 109 | 2 | 2 | 0 | 0 | 4 |
| 707 | 1 | 161 | 3 | 2 | 2 | 2 | 9 |
| 713 | 2 | 186 | 4 | 1 | 1 | 3 | 9 |
| 715 | 1 | 143 | 0 | 4 | 2 | 3 | 9 |
| 721 | 1 | 103 | 2 | 2 | 2 | 1 | 7 |
| 722 | 1 | 160 | 4 | 3 | 1 | 1 | 9 |
| 723 | 1 | 159 | 4 | 4 | 4 | 3 | 15 |
| 724 | 1 | 101 | 2 | 3 | 0 | 2 | 7 |
| 733 | 1 | 183 | 3 | 4 | 4 | 3 | 14 |
| 736 | 1 | 81 | 1 | 3 | 1 | 1 | 6 |
| 738 | 1 | 73 |  |  |  |  |  |
| 739 | 1 | 138 | 4 | 3 | 1 | 1 | 9 |
| 740 | 1 | 95 | 4 | 1 | 1 | 2 | 8 |
| 741 | 1 | 125 |  |  |  |  |  |
| 742 | 1 | 165 | 3 | 3 | 3 | 3 | 12 |
| 743 | 1 | 141 | 2 | 2 | 2 | 3 | 9 |
| 745 | 1 | 182 | 4 | 3 | 2 | 3 | 12 |
| 747 | 1 | 207 | 4 | 3 | 2 | 3 | 12 |
| 748 | 1 | 91 | 3 | 2 | 1 | 3 | 9 |
| 749 | 1 | 149 | 2 | 3 | 2 | 3 | 10 |
| 751 | 1 | 91 | 2 | 4 | 2 | 1 | 9 |
| 752 | 1 | 162 | 4 | 3 | 3 | 3 | 13 |
| 755 | 1 | 113 | 4 | 4 | 4 | 3 | 15 |
| 760 | 1 | 108 | 2 | 2 | 0 | 2 | 6 |
| 779 | 1 | 188 | 4 | 3 | 1 | 3 | 11 |
| 781 | 1 | 77 | 3 | 4 | 3 | 2 | 12 |
| 782 | 1 | 120 | 2 | 2 | 2 | 2 | 8 |
| 783 | 1 | 142 | 2 | 2 | 2 | 3 | 9 |
| 784 | 2 | 116 | 2 | 2 | 0 | 1 | 5 |
| 787 | 1 | 176 | 4 | 3 | 0 | 3 | 10 |
| 788 | 1 | 134 | 3 | 3 | 3 | 3 | 12 |
| 803 | 1 | 138 | 2 | 2 | 1 | 2 | 7 |
| 804 | 1 | 177 | 4 | 4 | 4 | 3 | 15 |
| 812 | 1 | 121 | 2 | 4 | 1 | 2 | 9 |
| 813 | 1 | 174 | 3 | 3 | 3 | 3 | 12 |
| 822 | 1 | 186 | 4 | 2 | 4 | 3 | 13 |
| 825 | 1 | 107 | 3 | 0 | 1 | 3 | 7 |
| 826 | 1 | 124 | 4 | 2 | 1 | 3 | 10 |
| 828 | 1 | 97 | 3 | 2 | 3 | 3 | 11 |
| 831 | 1 | 128 | 3 | 3 | 2 | 3 | 11 |
| 833 | 1 | 211 | 3 | 3 | 3 | 3 | 12 |
| 835 | 1 | 185 | 4 | 4 | 2 | 3 | 13 |
| 837 | 1 | 127 | 3 | 3 | 1 | 1 | 8 |
| 839 | 1 | 127 | 4 | 2 | 2 | 2 | 10 |
| 844 | 1 | 121 | 2 | 3 | 1 | 3 | 9 |
| 845 | 1 | 125 | 3 | 3 | 0 | 3 | 9 |
| 848 | 1 | 153 | 3 | 3 | 3 | 3 | 12 |
| 853 | 1 | 103 | 2 | 2 | 1 | 3 | 8 |
| 854 | 2 | 221 | 4 | 4 | 4 | 3 | 15 |
| 856 | 1 | 109 | 1 | 4 | 3 | 2 | 10 |
| 863 | 2 | 136 | 2 | 0 | 0 | 2 | 4 |
| 865 | 1 | 113 | 2 | 3 | 3 | 1 | 9 |
| 866 | 1 | 87 | 4 | 1 | 1 | 3 | 9 |
| 867 | 2 | 85 | 1 | 3 | 4 | 3 | 11 |
| 869 | 2 | 122 | 2 | 4 | 4 | 0 | 10 |
| 870 | 1 | 89 | 1 | 1 | 1 | 3 | 6 |
| 871 | 1 | 192 | 4 | 3 | 2 | 3 | 12 |
| 875 | 1 | 109 | 1 | 2 | 4 | 3 | 10 |
| 878 | 1 | 161 | 4 | 2 | 2 | 3 | 11 |
| 879 | 1 | 165 | 4 | 3 | 3 | 2 | 12 |
| 883 | 1 | 182 | 1 | 2 | 0 | 2 | 5 |
| 884 | 1 | 135 | 1 | 4 | 1 | 2 | 8 |
| 885 | 1 | 103 | 2 | 2 | 2 | 3 | 9 |
| 886 | 1 | 152 | 4 | 1 | 3 | 3 | 11 |
| 888 | 2 | 201 | 4 | 3 | 4 | 2 | 13 |

N(male)=97 N(female)=17 N(missing)=6
